# Active Tumor-Targeting by Smart Nanocarriers: A Potential Promising Approach to Overcome the Hurdles of Conventional Cancer Treatments

**DOI:** 10.31557/APJCP.2021.22.5.1331

**Published:** 2021-05

**Authors:** Enam Alhagh Charkhat Gorgich, Parisa Arbabi, Houman Parsaie

**Affiliations:** 1 *Department of Anatomy, School of Medicine, Iran University of Medical Sciences, Tehran, Iran. *; 2 *Intensive Care Unit, Mofarah Hospital, Tehran, Iran. *

**Keywords:** Cancer, tumor targeting, tumor microenvironment, nanoparticles, multifunctionalized

**Dear Editor**

Cancer is one of the most momentous and intricate life-threatening health problems which despite routine and combination treatments are still accompanied by high mortality worldwide (Gorgich et al., 2017). Because tumor cells have rapid and uncontrolled proliferation compare to normal cells, they need more nutrients, which in turn for supplying it begins to alter normal cellular signaling pathways and extensive angiogenesis in the specific tumor microenvironment (TME). During tumorigenesis, tumor cells undergo intra/extracellular specific biological changes as a result of increased metabolic requirements. These changes lead to overexpression of cell surface receptors, increased membrane channels and transporters (Heidari et al., 2017; Ancey et al., 2018). Based on these facts, diagnostic and therapeutic strategies can be designed for tumor-cells targeting.

Standard treatment of most tumors, particularly in the advanced stages, is based on multi-drug chemotherapy. Although this method initially provides considerable antitumor effects, in tumor progression, but often in continuation with the advent of limitations they are incapable of treating the tumor and can only increase the survival rate of patients. The most important hurdles of conventional treatment of tumors include physicochemical properties of drugs (poor solubility, high systemic toxicity and etc.), tumor heterogeneity, various tumorigenesis mechanisms, specific TME (pH, temperature and etc.), the presence of natural and tumor barriers, and interstitial tissue pressure in passive-tumor targeting method (Florea and Büsselberg, 2011; Bluthgen and Besse, 2015).

One promising strategy to overcom these obstacles is to use smart nanocarriers that have been approved by the FDA.

Recently, Park et al., (2021) designated a pH-sensitive multi-drug liposomes functionalized with folate receptor β (FRβ) for treatment of non-small cell lung cancer (NSCLC). They concluded that this platform with the ability to carry several drugs simultaneously, influence different aspects of tumorigenesis, with the least cytotoxic effects on off-target tissues and effective drug delivery to optimized dose. In addition, because of the use of pH-sensitive liposomes conjugated with FRβ which overexpressed in NSCLC cells, they directly targeted the TME and the tumor cells, respectively. 

In another study, researchers developed a smart liposomes-based delivery system for the treatment of gliomas. Their findings showed that dual therapy (temozolomide and bromodomain inhibitor) by smarted liposomes with transferrin, effectively exerts therapeutic effects on different aspects of tumors with desirable dosing and reduces tumor burden up to 2-fold, compared to conventional therapy in tumor-bearing mice. Moreover, due to the utilization of targeted liposomes, cellular uptake of liposomes dramatically increases, which in turn protects non-target tissues from the systemic drug cytotoxicity (Lam et al., 2018). Thus, by designing this nano platform, they overcame many of the limitations of conventional drug delivery in the central nervous system (Lam et al., 2018; Moradi et al., 2019). 

Hong et al., (2020) findings indicated that ginsenoside Rh2-liposomes with multifunctional properties, effectively accumulate in tumor site and increase cellular uptake via interaction between Rh2 and glucose transporter of 4T1 breast tumor cells. Furthermore, Rh2-liposomes had a significant effect on controlling cancer progression by influencing the characteristics of the TME via remodel of the tumor structure and modulating immune responses.

According to the above-aforementioned, the application of targeted nanoparticles in accordance with the specific TME in each tissue can increase cellular uptake by target cells, thereby increasing the therapeutic efficiency and reducing toxicity in off-target tissues. Therefore, tumor-targeting by smart nanocarriers can be used as an ideal potential promising strategy to bypass the obstacles of conventional cancer treatments and even isolation of tumor cells.
